# Development of a Classical Conditioning Task for Humans Examining Phasic Heart Rate Responses to Signaled Appetitive Stimuli: A Pilot Study

**DOI:** 10.3389/fnbeh.2021.639372

**Published:** 2021-04-01

**Authors:** Alessandra Sayão, Heloisa Alves, Emi Furukawa, Thomas Schultz Wenk, Mauricio Cagy, Samantha Gutierrez-Arango, Gail Tripp, Egas Caparelli-Dáquer

**Affiliations:** ^1^Lab of Electric Stimulation of the Nervous System Rio de Janeiro State University, Rio de Janeiro, Brazil; ^2^Psychology Department, University of Massachusetts Dartmouth, North Dartmouth, MA, United States; ^3^Okinawa Institute of Science and Technology Graduate University, Okinawa, Japan; ^4^Alberto Luiz Coimbra Institute for Graduate Studies and Research in Engineering, Federal University of Rio de Janeiro, Rio de Janeiro, Brazil; ^5^MIT Center for Extreme Bionics, Massachusetts Institute of Technology, Cambridge, MA, United States

**Keywords:** appetitive conditioning, cardiac deceleration, orienting response, attention, motivation

## Abstract

Cardiac responses to appetitive stimuli have been studied as indices of motivational states and attentional processes, the former being associated with cardiac acceleration and latter deceleration. Very few studies have examined heart rate changes in appetitive classical conditioning in humans. The current study describes the development and pilot testing of a classical conditioning task to assess cardiac responses to appetitive stimuli and cues that reliably precede them. Data from 18 adults were examined. They were shown initially neutral visual stimuli (putative CS) on a computer screen followed by pictures of high-caloric food (US). Phasic cardiac deceleration to food images was observed, consistent with an orienting response to motivationally significant stimuli. Similar responses were observed to non-appetitive stimuli when they were preceded by the cue associated with the food images, suggesting that attentional processes were engaged by conditioned stimuli. These autonomic changes provide significant information about classical conditioning effects in humans.

## Highlights

- A classical conditioning task assessed cardiac responses to appetitive US and CS
- Phasic cardiac deceleration to food images was observed
- Phasic cardiac deceleration to the US likely represents an orienting response
- Cardiac deceleration to the neutral stimuli was observed when preceded by CS+

## Introduction

Cardiac responses have been studied as indices of both motivational/emotional states and attentional processes (Hunt and Campbell, 1997; Bradley, 2009). Heart rate has been shown to accelerate in the presence of rewarding stimuli, such as monetary incentives during a motor task (Fowles et al., 1982), a plate of preferred food being available to look at, smell and then eat (Nederkoorn et al., 2000), repeated presentations of positive food images (Kuoppa et al., 2016), and anticipating the opportunity to perform a task to earn money (Rakover and Levita, 1973). Such acceleration is thought to reflect autonomic arousal and a heightened emotional state triggered by the presence of motivationally significant stimuli and mediated by sympathetic nervous system activation (Fowles et al., 1982; Richter, 2012). In contrast, deceleration of heart rate is observed in response to sensory stimuli (Graham and Clifton, 1966), and this is exaggerated when the stimuli are novel (Tuber et al., 1985; Gianaros and Quigley, 2001; Bradley, 2009) and have motivational or emotional significance (Abercrombie et al., 2008; Bradley et al., 2012). Such deceleration responses are thought to indicate cardiac orienting responses (Binder et al., 2005; Bradley, 2009), reflective of the organisms' attention to environmental stimuli and mediated by the parasympathetic system (Graham and Clifton, 1966; Barry, 1983).

To further examine the relationship between cardiac responses and behavior, recent human experimental studies have measured heart rate changes in response to reward or feedback during learning and decision making tasks (Somsen et al., 2000; Drobes et al., 2001; Crone et al., 2004; Groen et al., 2007; Luman et al., 2007; Kube et al., 2016; Kastner et al., 2017). The majority of these studies show cardiac deceleration to anticipation or receipt of incentives, pleasant stimuli, or positive feedback. In some studies, the deceleration occurred after the presentation of cues predicting reward/feedback (Kube et al., 2016; Kastner et al., 2017), in others immediately before (Jennings et al., 1992; Crone et al., 2004; Veen et al., 2004; Groen et al., 2007; Luman et al., 2007) and/or after (Somsen et al., 2000; Drobes et al., 2001; Luman et al., 2007) the reward/feedback onset. These responses have variously been hypothesized to represent reward/feedback expectancy, processing or evaluation. However, it is difficult to determine the extent to which these heart rate changes represent a motivational response. Heart rate changes have also been observed in response to task difficulty (Eubanks et al., 2002), attentional demands (Somsen et al., 2000), and motor response preparation (Rakover and Levita, 1973).

In these human studies, the involvement of the central nervous system is inferred in the observed cardiac deceleration responses. In parallel with the peripheral indications of attention, the central nervous system shows changes related to orientation. The central states, and corresponding orienting responses, however, are influenced by a number of factors, such as the context in which stimuli are presented, sensory features of stimuli, ongoing motor activity and affective state (Gianaros and Quigley, 2001). In addition to the direct autonomic control of cardiac functions, the basal state of the autonomic nervous system as well as its responses to stimuli affect the central nervous system inputs to heart rate changes. Similar challenges in identifying the origins of physiological responses have been encountered in human neuroimaging studies that examined the neural correlates of reward and feedback. To measure reward-related blood-oxygen-level-dependent (BOLD) responses unconfounded by motor responses or complex cognitive processes, researchers have employed classical conditioning paradigms (Bray and O'Doherty, 2007; Prévost et al., 2013; Furukawa et al., 2014, 2020; Klucken et al., 2016).

Classical conditioning refers to a process where previously neutral stimuli acquire motivational significance [becomes a conditioned stimulus (CS)] when paired repeatedly with a pleasant/rewarding stimulus or aversive stimulus [i.e., unconditioned stimulus (US)]. The CS then comes to elicit physiological and behavioral responses [conditioned responses (CR)] (Cartoni et al., 2016). The CR may be similar to the response [unconditioned response (UR)] elicited by appetitive or aversive stimuli (US), but this is not necessarily the case (Bourne, 1990). In appetitive conditioning, the US used is a pleasant/rewarding stimulus, a CS+ signals the occurrence of an appetitive US. To date, there have been very few studies examining heart rate changes in appetitive classical conditioning in humans. One study demonstrated cardiac acceleration to a tone after being repeatedly paired with delivery of a glucose solution to infants (Clifton, 1974). Another study with adult subjects reported cardiac deceleration in response to an abstract image that was repeatedly paired with display and receipt of preferred snacks (Prévost et al., 2012). Variation in the direction of heart rate change, as a CR, may reflect differences in cognitive development or autonomic system maturity. The acceleration in infants may reflect autonomic arousal triggered by CS+. Cardiac deceleration in adults may indicate cognitive awareness that reward will follow the CS+, leading to an orienting response.

A number of animal studies have measured phasic heart rate changes during appetitive classical conditioning paradigms (e.g., Harris and Brady, 1974; Cohen and Obrist, 1975; Randall et al., 1985; Kalin et al., 1996; Hunt and Campbell, 1997). The direction of heart rate changes in these studies has been mixed. Several studies showed cardiac acceleration in response to CS+ (Goldstein et al., 1970; Powell and Milligan, 1975; Randall et al., 1985; Boivin, 1986; McLaughlin and Powell, 1999). This acceleration was interpreted as a CR (Randall et al., 1985; Hunt and Campbell, 1997). Other studies showed cardiac deceleration in response to appetitive CS (Powell and Milligan, 1975; Campbell and Ampuero, 1985; Hunt and Campbell, 1997; McLaughlin and Powell, 1999), which was interpreted as a learned attentional/orienting response (Powell and Kazis, 1976; Campbell and Ampuero, 1985; Kapp et al., 1992). These varied outcomes have been attributed to paradigm differences, including reward type, as well as species differences in how sympathetic and parasympathetic nervous systems are activated (Hunt and Campbell, 1997; Roberts and Clarke, 2019).

Clearly, further human studies are needed to clarify the nature of cardiac responses to appetitive US and CS+ during classical conditioning. Procedural constraints have likely limited research to date, especially difficulties surrounding the use of food or liquid outcomes and deprivation requirements to ensure their continued salience during conditioning (Freeman et al., 2015; Manglani et al., 2017; Schad et al., 2019a). An appetitive classical conditioning paradigm that does not require food/liquid consumption would expand the opportunities to study heart rate changes to appetitive US and CS across a wide range of populations.

In this pilot study, we developed an appetitive classical conditioning task to assess (1) phasic heart rate responses to an US during a training and a subsequent test phase, and (2) phasic heart rate responses to the CS during the test phase when conditioning was expected to have taken place. In the task, participants were shown initially neutral visual stimuli (intended CS+) on a computer screen followed by pictures of high-caloric food (US). Pictures of food have been used as positive stimuli that elicit cardiac changes (Beaver et al., 2006; Pursey et al., 2014; Kuoppa et al., 2016).

We hypothesized heart rate changes in response to the US during the training phase. If the food pictures evoke a positive motivational state, heart rate acceleration would be expected. Pleasant and rewarding stimuli are associated with increases in sympathetic activity (Pribram and McGuinness, 1975; Braesicke et al., 2005; Critchley, 2009; Rudebeck et al., 2014) as well as behavioral activation (Schad et al., 2019b; Watson et al., 2019; da Costa et al., 2020). On the other hand, if the heart rate changes in response to food pictures are reflective of attentional/orienting responses, then deceleration would be expected.

During the test phase, after establishment of the CS-US association (conditioning), we hypothesized that the phasic heart rate changes would be observed in response to the CS. If the CS has acquired the motivational significance of the US, we would expect an acceleration of heart rate. Heart rate changes to the US may not be as pronounced during this phase. In animals, striatal dopamine responses to CS after conditioning are accompanied by diminished responses to US (Schultz et al., 1997; Pan et al., 2005). If the CS triggers the engagement of attentional processes, the heart rate deceleration to the CS would be expected. It is unclear whether the deceleration after the US onset would continue to be observed. Responses reflective of reward prediction errors may be observed when the US is not consistent with the CS preceded it (Kamin, 1967; Rescorla and Wagner, 1972).

## Materials and Methods

The study was approved by the ethics committee of the teaching hospital of the State University of Rio de Janeiro (UERJ) in Rio de Janeiro, Brazil. All participants were volunteers who provided written informed consent. At the beginning of each session, participants were informed that their heart rate would be recorded at rest and while they viewed some pictures.

### Subjects

Participants were 19 students attending UERJ, aged between 22 and 46 years. The inclusion criteria were normal or corrected vision, and no history of: psychiatric disorders; neurological diseases; cardiovascular diseases, including thoracic surgery or current use of cardiovascular medication; diabetes; obesity, defined as a BMI over 30 kg/m^2^. This information, together with demographic data, were collected via questionnaire. Following preliminary data analysis, one participant was excluded as an outlier, i.e., having significantly slower heart rate, compared to other participants. The final sample included data from eighteen participants^1^ (Table 1).

**Table 1:** Participant characteristics.

***N* = 18**	**Mean**	***SD***	**Range**
Age (years)	31.17	7.11	21–46
Weight (kg)	72.82	13.45	49–102
Height (cm)	168.88	8.59	154–185
Males *n* (%)	8 (44.4%)		

### Experimental Task

**Figure 1:**
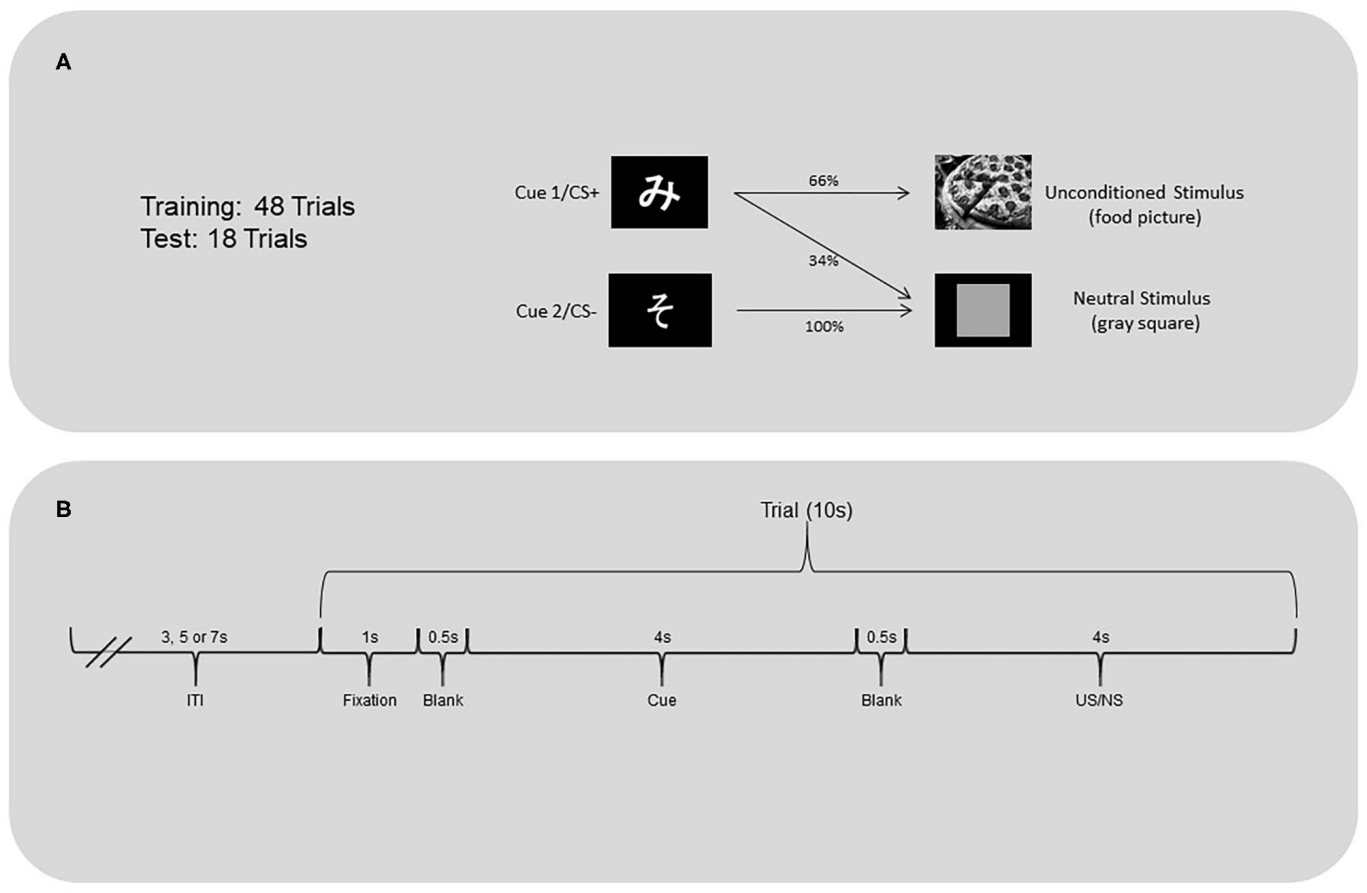
**(A)** Classical conditioning task and **(B)** trial timeline.

The appetitive classical conditioning task (Figure 1) was programmed using Delphi. Task stimuli were displayed on a 19-inch monitor screen (Dell 1901N). Each trial began with a fixation cross presented for 1 s followed by a 0.5 s black screen. One of two initially neutral stimuli (two Japanese hiragana characters unfamiliar to participants; Cue 1 or 2) was presented on a computer screen for 4 s followed by a blank screen for 0.5 s. After this, an appetitive unconditioned stimulus (US) or a neutral stimulus (NS) was presented for 4 s. The US were pictures of high-caloric food (e.g., brownies, pizza). These pictures have previously been rated by adults, of similar demographic backgrounds to the current participants, as significantly more likable than images of everyday objects (Furukawa et al., unpublished). The NS was an image of a gray square. Each US and NS was followed by a variable inter-trial-interval (3, 5, or 7 s), to reduce the temporal predictability of the next trial. No motor responses were required during the task.

The training and test phases of the task were programmed separately. During both phases, Cue 1 (CS+ after conditioning) was followed by the US 66% of the time and the NS 33% of the time. Cue 2 (CS- after conditioning) was always followed by the neutral stimulus. The training phase included 16 trials of Cue 1 followed by a US, 8 trials of Cue 1 followed by the neutral stimulus, and 24 trials of Cue 2 followed by the neutral stimulus. The test phase was implemented after completion of the training phase. This phase included six trials of Cue 1 followed by a US, three trials of Cue 1 followed by the neutral stimulus, and nine trials of Cue 2 followed by the neutral stimulus.

The number of presentations of Cue 1 and 2 was the same within each phase to ensure similar levels of habituation to the stimuli. The presentation order of the cues and US (22 different food images) and neutral stimuli were randomly scheduled and unique for each participant.

### ECG Recording

Prior to the experiment, two disposable adhesive electrocardiogram (ECG) electrodes were placed on participants' chest; one electrode on the left side of the sternum around the 5th intercostal space, and the other electrode near the right sternal border, around the 2nd intercostal space. The ECG signals were recorded on a Windows-based computer, with a Labview script, biopotential amplifier, sampling rate of 400 Hz, notch filter (60 Hz), high-pass filter cutoff set at 0.3 Hz, and low pass filter cutoff at 25 Hz. Before the experimental task began, baseline cardiac activity was recorded for 5 min at rest. The ECG recording was restarted and synchronized with the start of the experimental task. Testing was carried out in a quiet room. The experimenter was present for the duration of the experiment.

### Data Analysis

Data was extracted into and processed in Matlab. Phasic heart rate responses were estimated using R-R intervals. The R-R interval is the time between successive heartbeats.

To address normal heart rate variability within and across participants, the mean of the four R-R intervals preceding the start of each trial (before presentation of fixation point) was calculated to provide a measure of pre-trial R-R interval^2^ (Figure 1). The changes from this pre-trial R-R interval, i.e., “heart index” (iH), were then obtained by dividing each target R-R interval by the pre-trial R-R interval + the target R-R interval. For example, the interval between the first R peak and second R peak immediately following the US, divided by the pre-trial R-R interval plus the interval of the first and second R peaks, was the first post-US iH. An iH = 0.5 would indicate no change from the pre-trial heart rate. An iH value >0.5 would indicate cardiac deceleration (larger interval length), while iH value smaller than 0.5 would indicate cardiac acceleration (smaller interval length). The absolute values of iH indicated the magnitude of the phasic heart rate changes.

**Figure 2:**
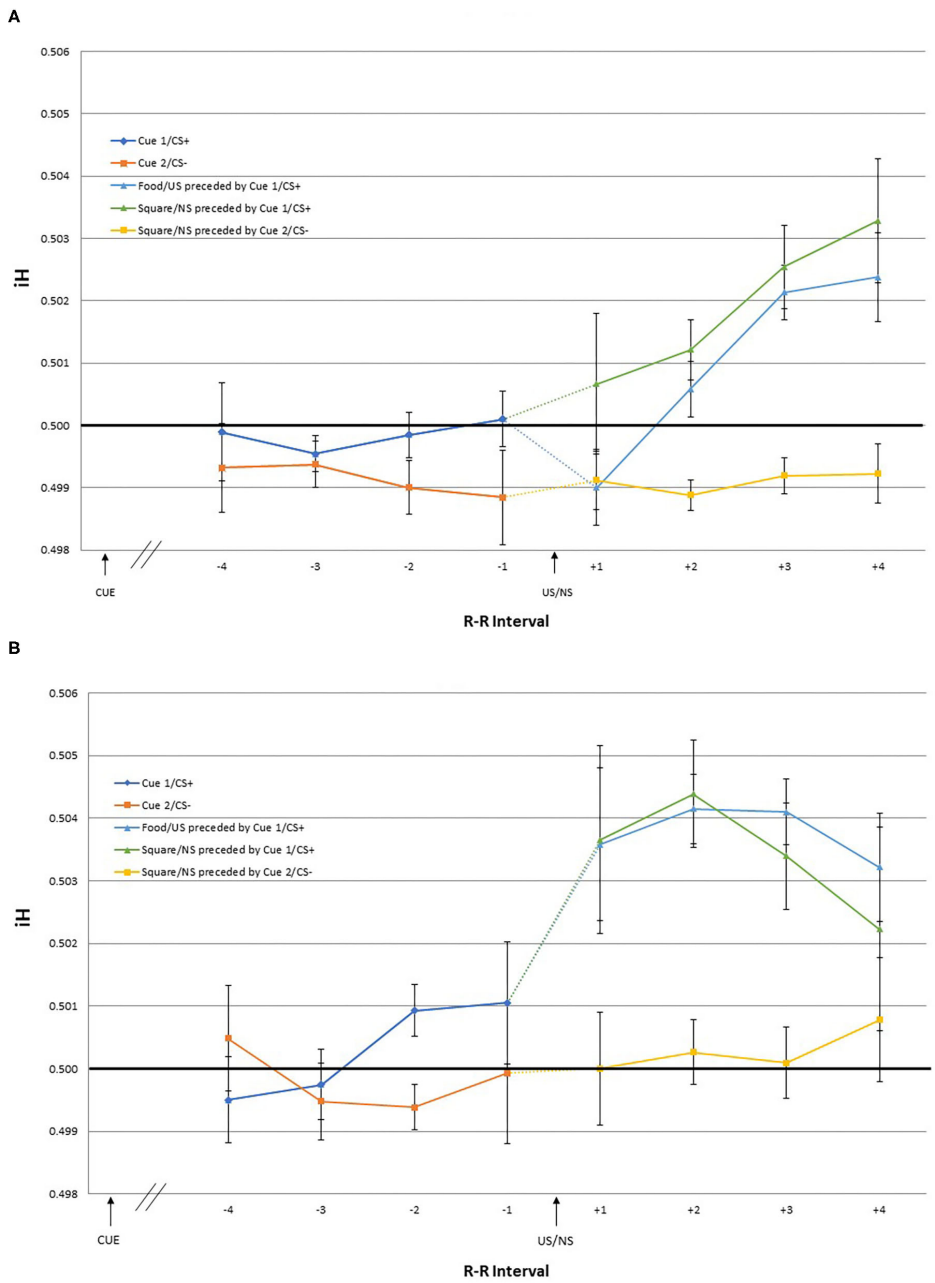
The mean iH values and within-subject standard errors for the first through fourth interval before US onset [separated by preceding cue type: Cue 1/CS+ (blue) and Cue 2/CS– (orange)], and the first through fourth interval after US onset [separated by the cue and US type; Food/US preceded by Cue 1/CS+ (light blue), Square/NS preceded by Cue 2/CS+ (green), and Square/NS preceded by Cue 2/CS– (yellow)] during the **(A)** training and **(B)** test phase.

**Figure 3:**
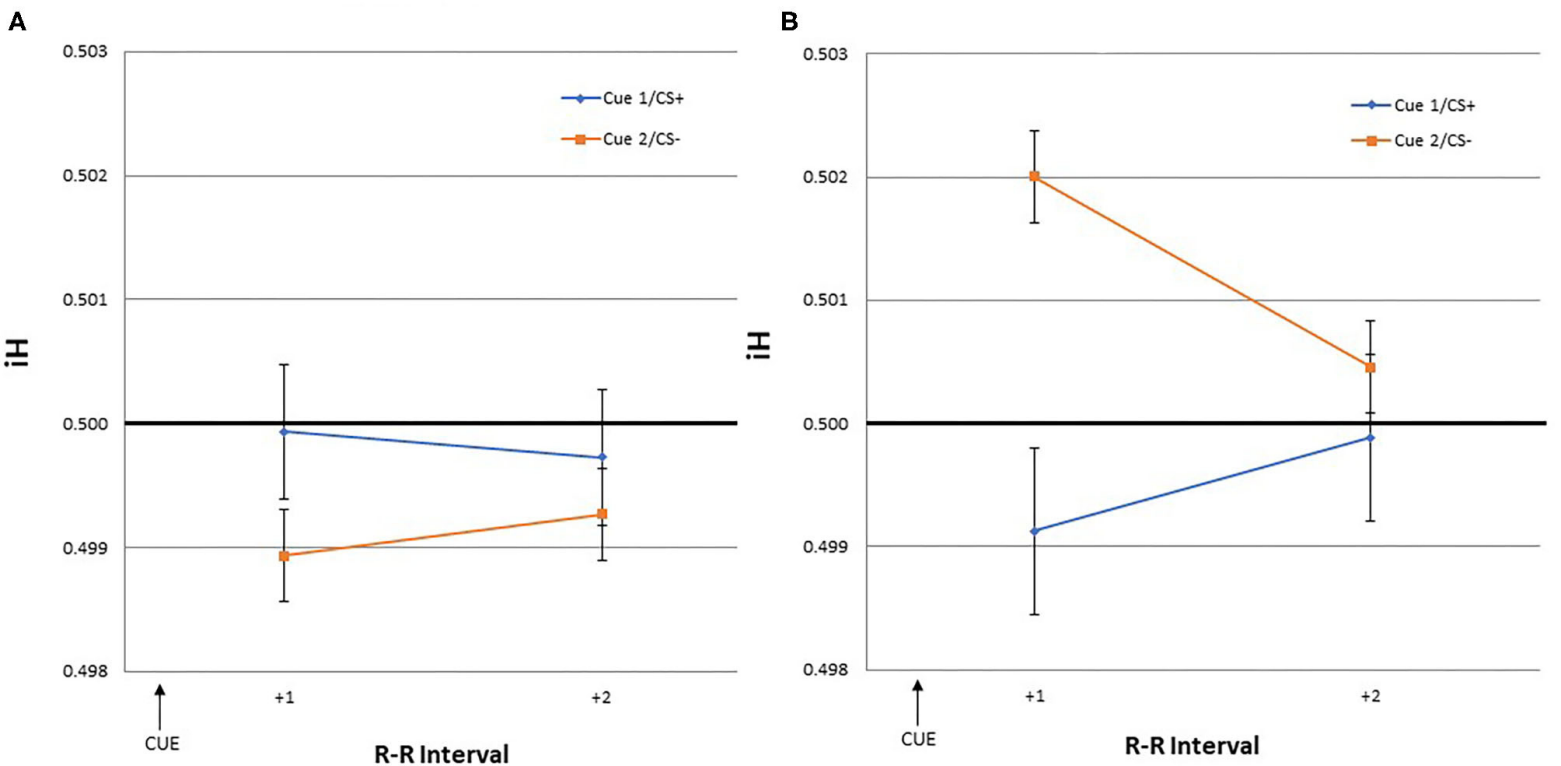
The mean iH values and within-subject standard errors for the first and second interval after a cue onset [Cue 1/CS+ (blue) or Cue 2/CS– (orange)] during the **(A)** training and **(B)** test phase.

The dependent variables were heart rate responses to the US/NS as well as to the cues that predicted them. The iH values were calculated for the first, second, third, and fourth R-R intervals following the US/NS presentation (interval +1, +2, +3, +4 after US/NS in Figure 2). As heart rate changes may be observed in anticipation of the stimulus presentation (Groen et al., 2007; Poli et al., 2007), the iH values were also calculated for a period before the US/NS presentation. The interval between the two R peaks immediately before the US/NS is the first pre-US/NS iH and so on (intervals −1, −2, −3, −4 before US in Figure 2). Similar procedures were followed to examine heart rate responses to cues, the iH values for the two intervals following Cue 1 and 2 were evaluated (interval +1, +2 after cue in Figure 3)^3^.

For each of the post-cue and pre-US/NS intervals, average iH values were obtained for each participant separately for two trial types: those following a Cue 1/CS+ presentation, and those following a Cue 2/CS– presentation. For each of the post-US/NS intervals, average iH values were obtained for three trial types: those following US preceded by Cue 1/CS+, those following the NS preceded by Cue 1/CS+, and those following the NS preceded by Cue 2/CS–.

Two-way repeated measures linear effects of time, US/NS and cue type on the iH values were examined using GLM. To explore the timing of the heart rate changes, one-sample tests were conducted to examine whether the iH value for each interval was significantly different from 0.5. All statistical analyses were undertaken with SPSS.

As tonic heart rate is reported to change in the presence of rewarding stimuli (Rakover and Levita, 1973; Fowles et al., 1982; Blanchard et al., 2000; Nederkoorn et al., 2000; Starcke et al., 2018), we undertook a series of exploratory analyses to test for these effects in our data. Tonic heart rate changes (bpm; beats per minute) and heart rate variability (RMSSD; the root mean square of successive differences between heart beats) during the rest period before the experimental task began and during the training and test phase were examined.

## Results

The participants' ratings of the food pictures, on a 4-point scale, completed after the experimental task, showed a similar level of likability (*M* = 3.28, *SD* = 0.75) as previously identified (*M* = 2.78, *SD* = 0.43) (Furukawa et al., unpublished).

### Motivational vs. Orienting Effects of US on Cardiac Responses

Heart rate changes after US (vs. neutral stimulus [NS]) were evaluated to examine whether the changes are reflective of motivational state (cardiac acceleration) or orienting responses (cardiac deceleration). This was done separately for the training and test phases.

Two-way repeated measures linear effects of time (four intervals), US/NS type (US, NS preceded by Cue 1/CS+, and NS preceded by Cue 2/CS-) and the interaction, were examined using GLM. During the training phase, there was a significant time^*^US/NS type interaction [*F*_(1, 17)_ = 6.58, *p* = 0.020] and a significant main effect of time [*F*_(1, 17)_ = 8.87, *p* = 0.008]. *Post-hoc* pairwise comparisons indicated that the mean iH values for the +1 and +2 intervals were significantly different from those of +3 and +4 intervals. Visual inspection of the data suggest that the significant effect of time is driven by heart rate changes following the US and NS preceded by Cue 1/CS+, while the iH values following the NS preceded by Cue 2/ CS– remained stable across the four intervals. During the test phase, no significant effects were observed.

Given the significant interaction effect observed in the GLM during the training phase, one-sample t-tests were conducted to examine for which intervals iH values were different from pre-trial R-R interval (i.e., different from 0.5) following the presentation of US and NS. Following the US, the significant effects were observed for +4 [*t*_(17)_ = 2.16, *p* = 0.046] interval, indicating cardiac deceleration. Differential results were obtained for the iH values following the NS depending on the preceding cue type. When the NS was preceded by Cue 1/CS+, the iH values after the NS were also significantly different from the pre-trial R-R interval for the +4 [*t*_(17)_ = 2.35, *p* = 0.031] interval, indicating deceleration. In contrast, when the NS was preceded by Cue 2/CS–, the iH values were not significantly different from the pre-trial heart rate.

While no significant effect was observed in the GLM for the test phase, to explore whether the pattern of heart rate changes observed during the test phase were consistent with that of the training phase, one-sample *t*-tests were conducted also for the iH values for the intervals during the test phase. One-sample *t*-tests showed that following the US presentation, the iH values were significantly different from the pre-trial R-R interval for the +2 [*t*_(17)_ = 2.34, *p* = 0.032], and +3 [*t*_(17)_ = 2.29, *p* = 0.035] intervals, indicating cardiac deceleration. Following the NS preceded by Cue 1/CS+, the iH values showed a trend toward deceleration for the +1 [*t*_(17)_ = 1.87, *p* = 0.079] and +2 [*t*_(17)_ = 1.93, *p* = 0.070] intervals. As in the training phase, the iH values following the NS preceded by Cue 2/CS– were not significantly different from the pre-trial R-R interval.

Analysis of the pre-US heart rate, during both the training and test phases, showed that no effect of time or preceding CS type, or the interaction. The iH values immediately prior to the US or NS presentation following Cue 1/CS+ (anticipation of a positive outcome) were not significantly different from the pre-trial R-R interval across all four intervals. Similarly, the iH values immediately prior to the NS presentation following Cue 2/CS– (anticipation of a negative outcome) were not significantly different from the pre-trial across all four intervals.

### Cardiac Responses to the Cues

The iH values for the two intervals following Cue 1/CS+ and Cue 2/CS– were evaluated during both the training and test phases. This was done to assess if similar cardiac responses to the US would occur in response to the cues predicting the US.

GLM yielded no significant effect of time, CS type, or the interaction. During the training and test phase, none of the iH values for the two intervals following the cue stimuli were significantly different from the pre-trial R-R interval.

### Tonic Heart Rate and Heart Rate Variability at Rest and During the Task

Repeated measures ANOVA indicated that bpm and RMSSD during the rest, training, and test phases were not different from each other, indicating no significant changes in the tonic heart rate or heart rate variability.

## Discussion

The current study describes the development and pilot testing of an appetitive classical conditioning task to assess heart rate responses to appetitive stimuli and cues preceding them in humans. Cardiac deceleration to US (food images with high likability ratings) was observed, consistent with an orienting response to motivationally significant stimuli. Similar cardiac responses were observed to the neutral stimuli (gray square) when participants were expecting the food images, i.e., following the cue typically associated with these images. Tonic heart rate and heart rate variability were not influenced by the task.

Significant changes in the heart rate responses following the US onset were observed during the training phase. Cardiac deceleration was observed for the fourth R-R interval, but not for the first through third intervals, following the stimulus presentation. Heart rate responses during the four intervals following the US onset did not change significantly during the test phase. Exploratory analyses showed that cardiac deceleration occurred earlier during the test phase, i.e., for the second and third R-R intervals, with effect beginning in the first interval. The effects of the cue might be seen in the latency of these cardiac responses from the US onset for the training vs. test phases. The cue proceeding the food images had developed predictive power and become a CS+, i.e., participants expected the food images following the cue presentation, which led to earlier heart rate changes.

Consistent with this hypothesis, the participants showed an orienting response to the neutral stimulus when it was preceded by the purported CS+, but not when it was preceded by the CS–. This may be taken as evidence that the cue had come to serve as a predictor of food pictures, a CS+, and therefore participants attended to the US regardless of its value. Alternatively, the uncertainty surrounding the CS+ maintained the salience of the stimuli that followed. The heart rate responses may be reflective of the prediction error (Kamin, 1967; Rescorla and Wagner, 1972), as the CS+ was predictive of the US only 66% of the time, whereas for the CS– was always associated with the neutral stimulus.

We hypothesized that increased sympathetic activity, and therefore heart rate acceleration, might be observed in response to the US (Pribram and McGuinness, 1975; Braesicke et al., 2005; Critchley, 2009). Increased autonomic arousal has been shown to be associated with positive motivational states in the presence of rewarding stimuli (Fowles et al., 1982; Richter, 2012). The results of the current study however showed clear cardiac deceleration to the presented appetitive stimuli. Food images were used as the US. The parasympathetic nervous system is activated when organisms digest food, which is accompanied by heart rate deceleration (Woods, 1972). This could explain the cardiac deceleration to the food images observed. Interestingly, other studies using non-classical conditioning tasks, such as learning and decision-making tasks, show phasic cardiac deceleration to non-food appetitive stimuli, such as monetary incentives, positive feedback, and pleasant pictures (Tuber et al., 1985; Somsen et al., 2000; Drobes et al., 2001). Heart rate deceleration is also seen in response to a range of aversive stimuli (Somsen et al., 2000; Drobes et al., 2001; Veen et al., 2004; Kuoppa et al., 2016; Kastner et al., 2017). These findings suggest that heart rate deceleration to the US observed in the current study is likely an attentional/orienting response. Heart rate deceleration assists organisms to attend selectively to stimuli important for survival (Hunt and Campbell, 1997).

Expectations created by the CS+ influenced heart rate responses to the US and the neutral stimuli in the current study. During the test phase, heart rate changes were observed with shorter latency from the US onset. When the neutral stimulus followed the CS+, cardiac deceleration was also observed. Together these results offer some evidence that the CS+ was eliciting the cardiac deceleration. Heart rate changes after feedback have also been suggested to reflect expectation errors (Somsen et al., 2000; Luman et al., 2007). The CS+ established an expectation that a positive stimulus would follow. However, the CS+ was not always followed by US. Under such conditions of uncertainty, the US itself provides meaningful feedback, eliciting orienting responses. In humans, cardiac responses may be linked to the cognitive processes of attending as well as to the valence of the stimulus (McLaughlin and Powell, 1999). It is interesting that the only human classical conditioning study demonstrating cardiac acceleration to a CS+ was with infants, whose cognitive skills are less developed (Clifton, 1974). In animal studies, the direction of the heart rate changes to the CS+ is mixed (Hunt and Campbell, 1997), with some reporting bi-phasic responses with initial orienting deceleration to the CS+ onset then cardiac acceleration closer to the time of US delivery (Holland, 1979).

The current task, together with the data processing and analysis methods, provide reliable measurements of heart rate responses during appetitive classical conditioning in humans. These reflect important developments toward a new methodology. Differential effects of US vs. neutral stimuli on heart rate were observed, and the data reflected awareness of the purported CS+ vs. CS–. As the task was simple and no behavioral responses were required, these cardiac responses were likely elicited by these stimuli, unconfounded by task difficulty (Richter, 2010; Fairclough and Ewing, 2017) or action/action preparation (Cooke et al., 2014; Rösler and Gamer, 2019). The data offer new information on adult human responses to appetitive stimuli and cues that predict them.

Despite the promise of this new methodology and the interesting findings obtained, the current study is not without limitations. As a pilot study, the sample size is relatively small, possibly impacting statistical significance of findings. The total number of trials may need to be expanded in future studies, during both training and testing to better detect effects. More trials/stronger associations would help clarify the importance of these trends. As presentation of trial type was randomized, each participant received trials in a unique order. This prevented us from examining the time course of the heart rate changes within the training and test phases. Moving forward we would recommend using the same trial order presentation across participants. It would also be helpful to present the US repeatedly without cues to examine whether the shorter latency between the US onset and the heart rate responses reflect participants becoming desensitized after the repeated US presentations; however, such desensitization would likely be associated with reduced heart rate changes. We would also consider including longer intervals, i.e., more heart beats, before and after presentation of the US to further clarify the nature of cardiac changes and their timing. There is some evidence that anticipatory heart rate changes are bi- or tri-phasic, responses following the CS changing as the time gets closer to the US onset (Rakover and Levita, 1973; Bohlin and Kjellberg, 1979). While food images are often used experimentally in human studies, we question if they served as strong appetitive stimuli in this study. Furthermore, we did not control for participants' hunger levels. In non-human studies, animals are usually food/liquid deprived, with mixed findings attributed to differing stimulus saliency (Hunt and Campbell, 1997; McLaughlin and Powell, 1999). The use of consumable stimuli and/or food/liquid restriction, as well as other types of appetitive stimuli, in future studies with the current task should be considered. If similar results are obtained using other appetitive stimuli, it would support the suggestion that the heart rate responses seen in the current study are reflective of appetitive conditioning rather than other forms of associative learning. If using consumable US, however, the effects of the act of consuming the food or drink on heart rate responses would need to be carefully considered. In any studies, in addition to the motivational and attentional processes, other factors that could affect heart rate, such as motor responses, stimulus type, presentation and timing, should be taken into account in hypothesis generation, task development and the interpretation of results. A final consideration for future studies is the extent to which the CS+ is predictive of the US. A task containing only CS+ trials may lead to different tonic heart rates during the rest, training, and test phases and show gradual heart rate acceleration with time.

Evaluating and monitoring autonomic changes during appetitive classical conditioning is important to understanding how predictive cues exert control over human behaviors. In this pilot study, a cue that typically preceded food images led to cardiac orienting responses. These data suggest that attentional processes can be engaged or disengaged by conditioned stimuli. Heart rate changes are relatively easily measured physiological responses that can be assessed across settings and populations. The study also generated helpful future considerations. Further studies employing larger samples of typically developing adults and children would clarify whether these responses are consistent across the life span. Individuals with clinical conditions may demonstrate different cardiac response patterns to cues and appetitive stimuli. If such differences can be relatively easily identified, i.e., using the current task and analytical methods, this information can be used to better understand the underlying nature of these disorders and potentially their management.

## Data Availability Statement

The data supporting the conclusion of this study are available upon reasonable request to the corresponding author.

## Ethics Statement

This study was reviewed and approved by Comissão de Ética em Pesquisa (Coep)—UERJ. The patients/participants provided their written informed consent to participate in this study.

## Author Contributions

AS, HA, and EC-D: participated in study conceptualization, data collection, and data analysis. EF and TSW: participated in study conceptualization and data analysis. MC: participated in experimental task development. SG-A: participated in data analysis. GT: participated in study conceptualization. All authors contributed to the article and approved the submitted version.

## Conflict of Interest

The authors declare that the research was conducted in the absence of any commercial or financial relationships that could be construed as a potential conflict of interest.

## Footnotes

^1^A total of 23 trials from eight participants were excluded due to unclear recordings (undefined peaks). Details available upon request.

^2^Four intervals were used as most participants' heart rate responses consistently had at least four intervals during the ITI. There was no significant difference in the resulting iHs when using two, three, or four intervals to calculate the pre-trial R-R interval. In addition, the pre-trial means were compared for the 3, 5, and 7 s ITI trials; no significant difference was observed.

^3^In most participants, there were 4–6 R-R intervals between the cue and US/NS. Thus, for some participants, the four pre-US/NS intervals and two post-cue intervals overlapped.
